# An Open-Source 3D–Printed Recording Stage with Customizable Chambers for Ex Vivo Experiments

**DOI:** 10.1523/ENEURO.0257-24.2024

**Published:** 2024-09-13

**Authors:** Preston C. Withers, Hunter J. Morrill, R. Ryley Parrish

**Affiliations:** ^1^Department of Cell Biology and Physiology, Brigham Young University, Provo, Utah 84602; ^2^Neuroscience Center, Brigham Young University, Provo, Utah 84602

**Keywords:** additive manufacturing, biomedical engineering, custom laboratory equipment, electrophysiology, neuroscience tools, spreading depolarization

## Abstract

Much of what has been discovered concerning neurophysiological mechanisms can be credited to ex vivo biomedical experiments. Beyond these discoveries, ex vivo research techniques have enhanced the global understanding of human physiology and pathology in almost every biomedical specialty. Naturally, ex vivo experiments are among the most desired methods of research, particularly in the field of neuroscience. Ex vivo experiment platforms may be purchased commercially. However, their substantial cost and sometimes limited availability can render them inaccessible to many research labs. Moreover, these manufactured systems are often rigid in function with no possibility of customization, severely narrowing their capabilities. However, developing essential components for ex vivo laboratory systems with a fused deposition modeling printer provides a practical solution to each of these obstacles. Here, we provide the designs and construction process for an easily accessible, highly adaptable recording stage with modifiable submersion chambers using a 3D printer for a total cost under $15.00. With the versatility afforded by the exchangeable custom chambers, the system may be used to conduct research on a variety of ex vivo tissue preparations, paving the way for novel research.

## Significance Statement

Ex vivo research techniques on animal models are critical to ongoing research in most medical fields, and much of what has been discovered concerning the physiology of neurons can be attributed to such experiments. As useful as commercially available designs have proven to be, they can prove difficult to access for some researchers due to their significant costs. Moreover, these systems are often restricted in their experimental capabilities and are unable to be modified. We propose a 3D-printable design that is readily available, incredibly affordable, highly adaptable, and completely customizable, a design capable of moving crucial research forward in meaningful ways.

## Introduction

Ex vivo experimental models have been instrumental in advancing research in many biomedical disciplines. These models have provided invaluable insight into many aspects of neurophysiology, including the cellular mechanisms of the neuronal synapse (Heuser et al., 1979; Martin et al., 2000; Siksou et al., 2007), memory formation (Collingridge et al., 1983; Kauer et al., 1988; Zakharenko et al., 2001), seizure initiation and propagation (Cammarota et al., 2013; Mackenzie-Gray Scott et al., 2022; Thouta et al., 2022), and spreading depolarization (Leao, 1947; Yao et al., 2011; Parrish et al., 2023)*.* Ex vivo models have also been applied in a variety of other research applications, including the study of ophthalmology (Liu and Wang, 2009; Bao et al., 2018; Schnichels et al., 2019), lung tissue diseases (Nicholas et al., 2015; Xia et al., 2023), intestinal infections (Grant et al., 2006; Taebnia et al., 2023), ossification (Staines et al., 2019; Miyamoto et al., 2021), intervertebral disk degeneration (Tang et al., 2022; Salzer et al., 2023), tumorigenesis (Kim, 2005; Pinto et al., 2020), and probiotics (Pagnini et al., 2016; Schmitter et al., 2018). Identified by their significantly lower costs and similar drug screening capabilities, ex vivo experiments are preferred over in vivo techniques in many research labs (Lossi and Merighi, 2018). Furthermore, ex vivo systems allow researchers to more easily replicate experiments while benefiting from additional control (Hamilton and Santhakumar, 2020; Vaughan et al., 2024). Challenges associated with studying ex vivo animal models can include high costs and limited availability of equipment. Moreover, commercially available designs are often specialized for specific experiments without options for customization, severely limiting versatility. Fortunately, 3D printing provides innovative solutions to each of these problems.

Fused deposition modeling (FDM) printers are capable of printing with several inexpensive filaments and are becoming increasingly accessible around the world. Available for as little as $350, FDM 3D printers are capable of producing quality custom laboratory parts with very low operational expenses (Coakley and Hurt, 2016). Furthermore, the operation of 3D printers can be performed by individuals with no engineering experience due to the expansive 3D printing community, tutorials, and intuitive software.

In neuroscience research labs, 3D-printed designs have been created to assist in the study of in vivo rodent models (Ben Youss et al., 2022), and independent in vitro recording chambers have been introduced without solution perfusion capabilities (Dondzillo et al., 2015). Additionally, various 3D-printed recording chamber designs have been developed with the capability of improving circulation and general function, supplemental to an existing ex vivo recording stage (Slater et al., 2015). However, no published designs exist for a completely 3D-printable ex vivo recording stage with the possibility of introducing custom recording chambers. Such a model introduces tremendous versatility by allowing for future designs specific to the needs of the research lab. Here, we introduce an ex vivo recording stage and chamber that can be customized and printed with minimal resources. This model has been designed with a focus on use-case adaptability and hassle-free tissue support with an added bonus of affordability.

## Materials and Methods

### Printer

Each of the designs in this paper was printed with a Taz 6 FDM printer (LulzBot), equipped with a Single Extruder v2.1 toolhead (LulzBot) and 1.75 mm polylactic acid (PLA) filament (Creality). The designs were created using Fusion360 (Autodesk) and exported as .stl files. The Cura LulzBot Edition 3.6 (produced based on Ultimaker open-source code) software was used to convert the .stl file into the geometric code using a custom “high detail” setting (in which the filament is printed with a low layer height to improve precision). The designs were then printed directly from a computer via a USB connection to the printer. As an alternative to connecting a computer directly to the printer, the geometric code may be downloaded onto an SD card, which may be inserted into the printer to initiate the printing process.

### Required materials to construct 3D-printed ex vivo recording stage and single chamber

**Figure 1.**
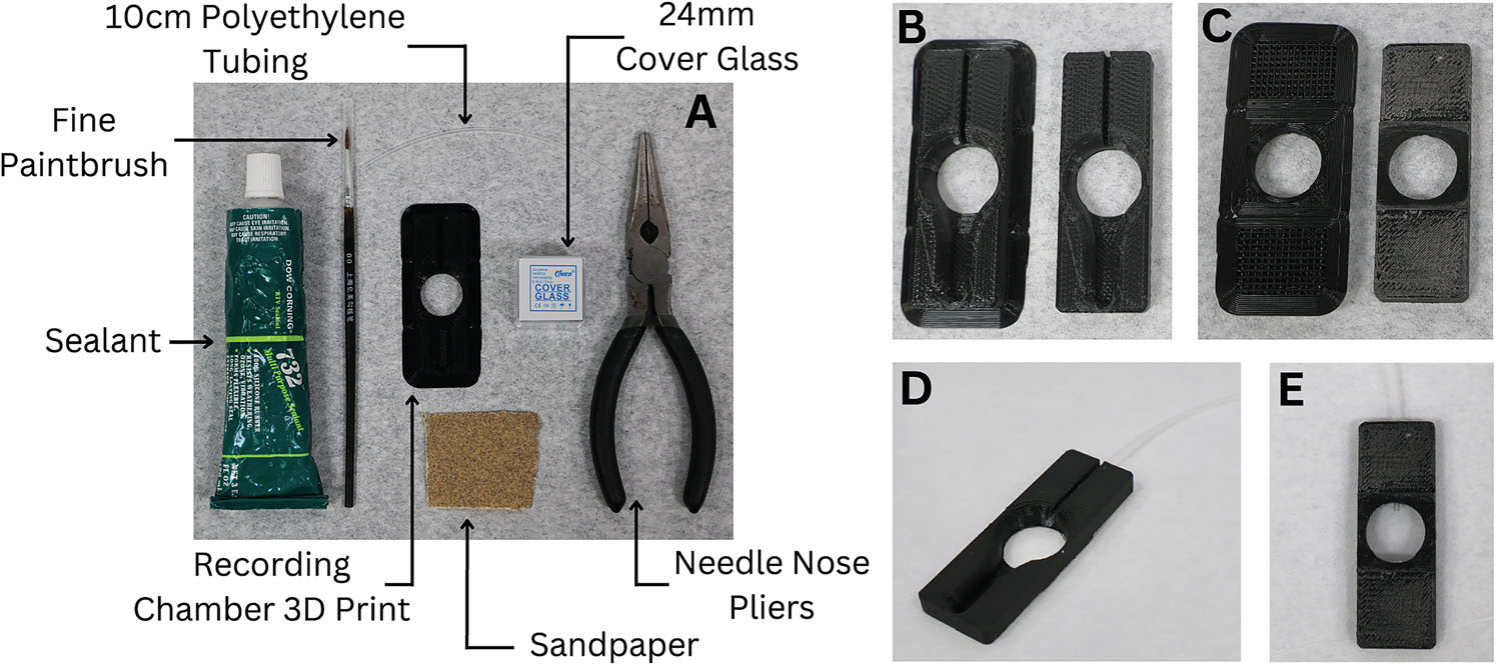
Materials needed and assembly of recording chamber. ***A***, The materials needed to construct 3D-printed recording chamber. ***B***, Top of recording chamber before and after removal of brim support. ***C***, Bottom of recording chamber before and after removal of brim and supports. ***D***, ***E***, Top and bottom, respectively, of finished recording chamber with polyethylene tubing and cover glass.

**Figure 2.**
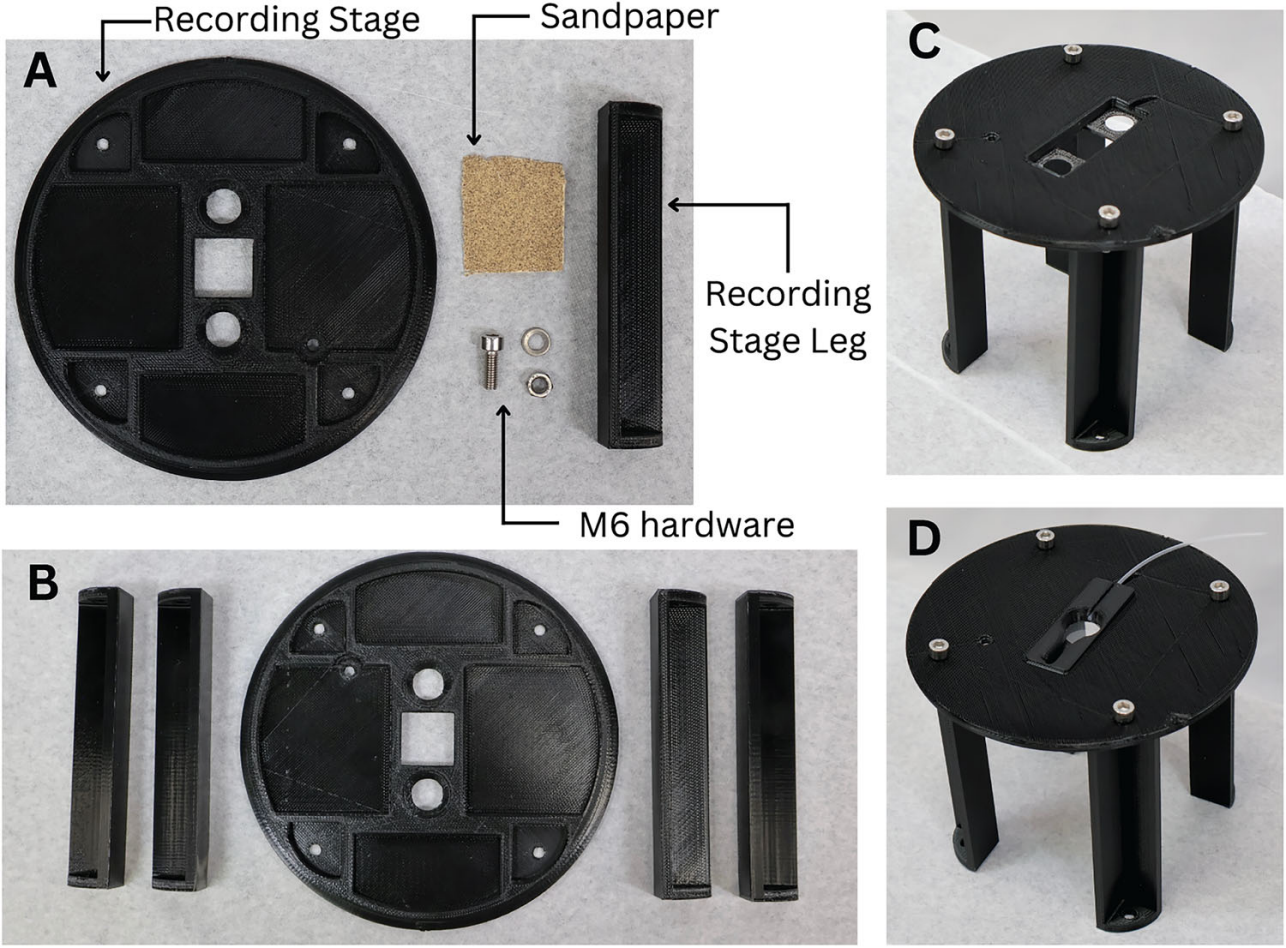
Materials needed and assembly of 3D-printed recording stage. ***A***, The materials needed to construct 3D-printed recording stage. ***B***, Recording stage components prepared for assembly with supports removed. ***C***, Recording stage assembled with M6 hardware. ***D***, Recording stage with recording chamber insert in place.

Table 1 outlines the required materials to construct a 3D-printed recording chamber (Fig. 1*A*) and the entire recording stage (Fig. 2*A*), complete with suggested vendors, the quantity of each item, and the price. The total of $14.70 (Table 1) offers a comparable product with commercially produced models, such as the Brain Slice Chamber-1: Interface and Submerged (BSC1IS; AutoMate Scientific) chamber, which is marketed at $2,959.

**Table 1.** The required materials to construct a 3D-printed recording chamber and the entire recording stage, complete with suggested vendors, the quantity of each item, and the price for the necessary quantity

Item description	Vendor	Quantity	Price
1.75 mm PLA	Amazon	351 g	$6.31
M6 20 mm flat screw	Cheap stair parts	1	$0.38
M6 10 mm screw	Accu	4	$2.40
M6 16 mm screw	Accu	4	$2.32
M6 nuts	Accu	5	$1.55
M6 washers	Accu	5	$1.65
24 * 24 mm coverslip	AmScope	1	$0.07
Polyethylene tubing	Thomas Specific	10	$0.02
Total for one system			$14.70

### Constructing the 3D-printed recording stage and single modifiable chamber

**Figure 3.**
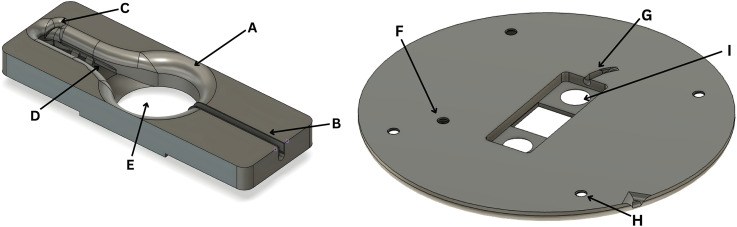
Example of possible recording chamber and stage features. ***A***, Wide brim for electrode access. ***B***, Slot for inflow tubing. ***C***, Notch for outflow access. ***D***, Channels for reference wires. ***E***, Oblong well for high flow rate perfusion. ***F***, Screw hole for magnetic outflow attachment. ***G***, Notch for inflow tubing. ***H***, Holes for attachment to legs. ***I***, Holes allowing for easy removal of recording chamber insert.

1. Using an FDM printer, print each of the recording stage components and the desired recording chamber (Extended Data Fig. 1-1).
  - Convert the .stl files (provided in the included ZIP folder) into geometric code with a “high detail” setting using a slicer software program such as Cura (Ultimaker).
  - With an FDM printer, print the recording stage and the desired recording chamber using the obtained geometric code and PLA filament. The printing time is estimated to be 18 h (17.33 h for the recording stage parts and 0.66 h for the recording chamber). However, the printing time will vary based on the 3D printer, filament diameter, and printer settings.
2. Prepare the printed parts. It is recommended to take these steps soon after printing each part for the best results. Figures 1 and 2 display how the recording chamber and the recording stage should appear respectively after these steps have been completed.
  - Remove the brim from each printed body with the needle nose pliers (Fig. 1*B*,*C*). Also, remove the support lattices from each printed part.
  - Sand the areas where the supports were removed until they are level. A file may be helpful.
3. Assemble the recording chamber.
  - With a razor blade, cut a 10 cm section of the polyethylene tubing. Cutting at an angle will make the tubing easier to insert into the connection tubing.
  - Using the paintbrush, apply glue to the inflow channel of the recording chamber where the channel drains into the main body (Fig. 3*B*). Being careful not to obstruct the tubing with glue, place the tube inside the channel with the flat end of the tube coming out of the channel by ∼2 mm toward the center of the chamber (Fig. 1*D*,*E*). The glue should form a tight seal around the tube in the channel.
  - On the bottom side of the stage guide piece, generously apply silicone to the square ledge around the bore with the paintbrush (Fig. 1*D*,*E*). Carefully place a glass coverslip found in Table 1 onto the square ledge where the glue was applied. Apply slight pressure to form a tight seal all the way around, and ensure the coverslip is flush with the edges. Allow 24 h for the glue to completely dry before use.
4. Assemble the microscopy stage.
  - Attach each of the legs to the microscopy stage with the 16 mm M6 screws, nuts, and washers (Figs. 2*C*, 3*H*).
  - If the flat M6 screw will be used to magnetically attach the recommended outflow piece, insert the screw into the hole next to the slot for the recording chamber (Fig. 3*F*), and secure it in place with the last washer and nut. If no flat M6 screws are available, you may glue a flat screw of a smaller size in place of the M6 screw.
  - Next, bolt the table legs to a tapped antivibration table using the 10 mm M6 bolts. Note that some antivibration tables may have tapped holes of different diameters or may not be tapped. Alternatively, the system can be secured to an untapped laboratory surface with double-sided high–quality Velcro.
5. Once the glue on the microscopy stage guide piece has dried, place the recording chamber inside the recording stage chamber slot (Fig. 3*I*).
6. Attach all accessory equipment (Fig. 5*B*).

10.1523/ENEURO.0257-24.2024.f1-1 Figure 1-1 **3D print and CAD files for the recording stage, recording chamber, and tissue holding chamber.** STL and F3D files for download, adjustment, and printing of the recording stage, recording chamber, and tissue holding chamber. Download Figure 1-1, ZIP file.

### Features of the 3D-printed recording chambers and recording stage

The included recording chamber and recording stage designs include features meant to make the system as versatile and convenient as possible, including each of the following (Fig. 3).

#### Chambers of varied depths

The provided chamber designs are optimized for the submersion of shallow ex vivo models (such as rodent brain slices in 2.4 to 5.4 mm of fluid) and deep ex vivo models (such as entire adult zebrafish brains with a depth of 3.9 to 7.0 mm). The shallower submersion chamber is not sufficiently deep to completely cover the zebrafish brain, and the deeper submersion chamber is much deeper than required for the rodent brain slice, distorting imaging, and decreasing perfusion across the tissue. The difference in the chamber depth is essential in allowing experiments to be effectively conducted on the tissue of varied heights. The fluid depth is further controlled by raising or lowering the outflow arm (Fig. 5*B*).

#### Outflow divot

The outflow divot provides easy access of the outflow tubing to the solution (Fig. 3*C*). By adjusting the outflow tube arm, the fluid depth may be quickly and more precisely controlled. When compounded with the increased freedom provided by the exchangeable chambers, the system has excellent fluid level control, which has proved beneficial in managing noise common to submersion systems with integrated perfusion (Dondzillo et al., 2015). This feature has also resulted in an observed improvement in image quality and the visualization of electrodes.

#### Disconnectable tubing

The recording chamber is designed to be easily removable from the recording stage and perfusion system for quick exchanges between chambers. The short tubing installed in the chamber (Figs. 1, 3*B*) can be inserted into the larger tubing of a perfusion system. This provides a better seal and eliminates the need for the tubing to be removed from the recording chamber each time the chamber is removed.

#### Channels and rings for reference wire

The recording chamber is equipped with channels for the reference wire bilaterally along the lower edges of the outflow channel (Fig. 3*D*). When prepared in a wider shape than the channel, the reference wire will press outward into these channels and remain in place. The rings provide further stability and support by eliminating the possibility of the wire being removed any way except for toward the inflow. When chambers are exchanged, the reference wire may easily be pulled out toward the inflow and reinserted into the channels and rings of the new chamber.

#### Wide brim for access

A wide sloping access area was developed to provide easy access to the model by multiple head stages, including at wide angles (Fig. 3*A*). The base circle has a diameter of 18.4 mm, and the highest point has a minimum circular access point with a diameter of 25.4 mm. In other words, the center of the chamber floor can be reached from the sides of the chamber by an angle as low as 30.6° from the horizontal for the deeper recording chamber and as low as 24.7° for the shallower recording chamber.

#### Recording stage outflow screw hole

Insertion of a flat screw allows for the secure magnetic attachment of outflow systems such as a Bath Perfusion Tool (Scientifica; Fig. 3*F*).

#### Recording chamber removal holes

The circular holes in the recording stage below the recording chamber allow for the easy removal of the recording chamber without touching the delicate glass of the coverslip (Fig. 3*I*).

### Required materials to construct 3D-printed holding chamber

Table 2 outlines the required materials to construct a 3D-printed holding chamber, complete with suggested vendors, the quantity of each item, and the price. The total of $0.58 (Table 2) offers a comparable product with commercially produced models, such as the Brain Slice Keeper 4 (AutoMate Scientific), which is marketed at $872.00.

**Table 2.** The required materials to construct a 3D-printed holding chamber, complete with suggested vendors, the quantity of each item, and the price for the necessary quantity

Item description	Vendor	Quantity	Price
1.75 mm PLA	Amazon	27 g	$0.49
Unbleached cheesecloth	Amazon	8.5 * 8.5 cm	$0.09
Total			$0.58

### Constructing the 3D-printed holding chamber

**Figure 4.**
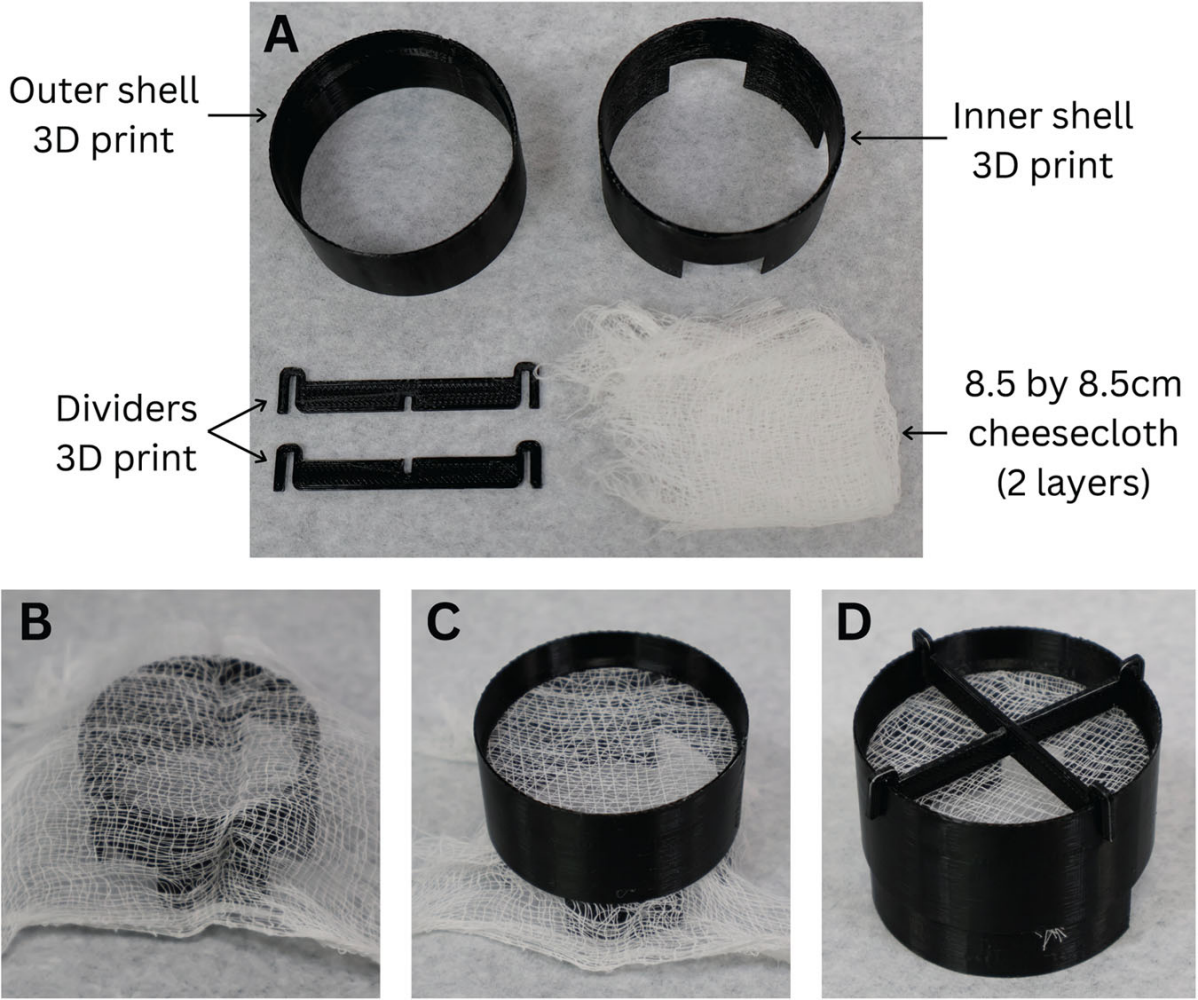
Materials needed and assembly of 3D-printed tissue holding chamber. ***A***, Required materials for 3D-printed tissue holding chamber. Cheesecloth can be substituted for a finer fabric for smaller tissue samples. ***B***, Inner shell with doubled-over cheesecloth. ***C***, Outer shell slid over inner shell with cheesecloth stretched between the layers. ***D***, Assembled holding chamber including dividers.

**Figure 5.**
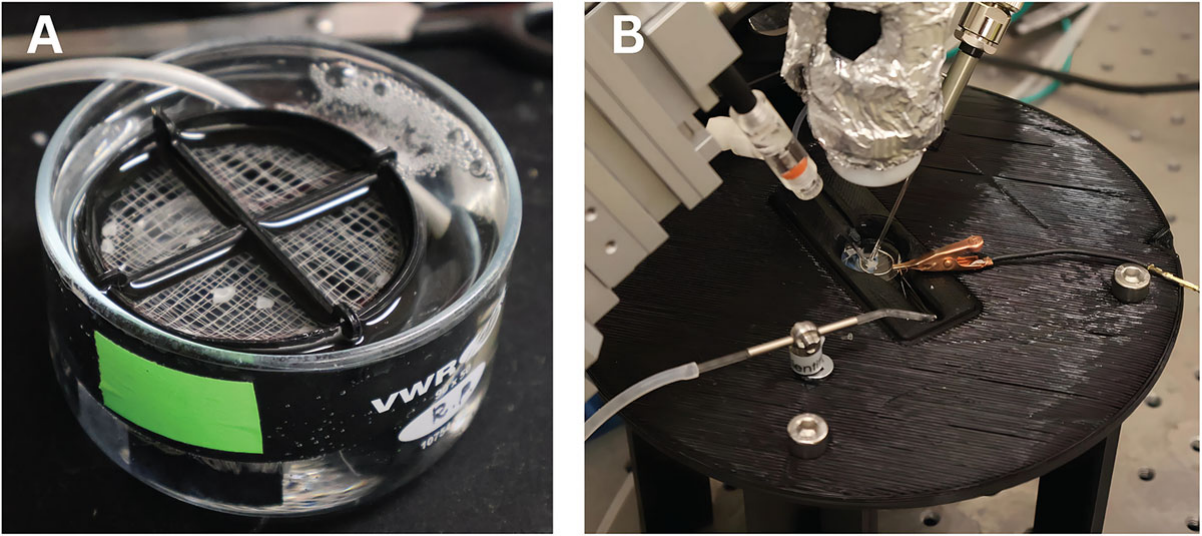
3D-printed holding chamber and stage in use for LFP recordings in a mouse neocortical tissue. ***A***, Holding chamber in use with carbogenated aCSF maintaining health of mouse brain slices. ***B***, Recording stage and chamber in use with perfusion system, reference electrode, LFP microelectrode, digital microscope, and picospritzer.

7. Using an FDM printer, print the holding chamber elements, including the inner chamber component, outer chamber component, and divider pieces (Fig. 4*A*).
  (a) Convert the holding chamber .stl files (provided in the included ZIP folder) into geometric code with a “high detail” setting using a slicer software program such as Cura (Ultimaker).
  (b) Print the holding chamber elements using the obtained geometric code and PLA filament. The printing time is estimated to be 3.38 h, with slight variations based on the printer, filament diameter, and printer settings.
8. Remove the brim from each printed part using the needle nose pliers (as displayed in Fig. 4*A*).
9. Spread a two-layered 8.5 * 8.5 cm section of the cheesecloth across the top of the holding chamber inner shell. Leg hosiery is another affordable option in place of cheesecloth (Fig. 4*B*).
10. Slide the outer holding chamber piece around the inner holding chamber piece down from the top, until the outer holding chamber ledge rests on the top of the inner holding chamber (Fig. 4*C*).
11. Cut any exposed cheesecloth with scissors or a razor blade.
12. Place the holding chamber in a small beaker with a solution level just above the cheesecloth. Place the carbogen stone on the outside of the largest leg of the holding chamber to prevent the brains or brain slices from direct exposure to bubbles (Fig. 5*A*).
13. Place the dividers on top of the outer holding chamber piece, if desired, to divide the chamber in half or quarter sections (Fig. 4*D*).

## Results

**Figure 6.**
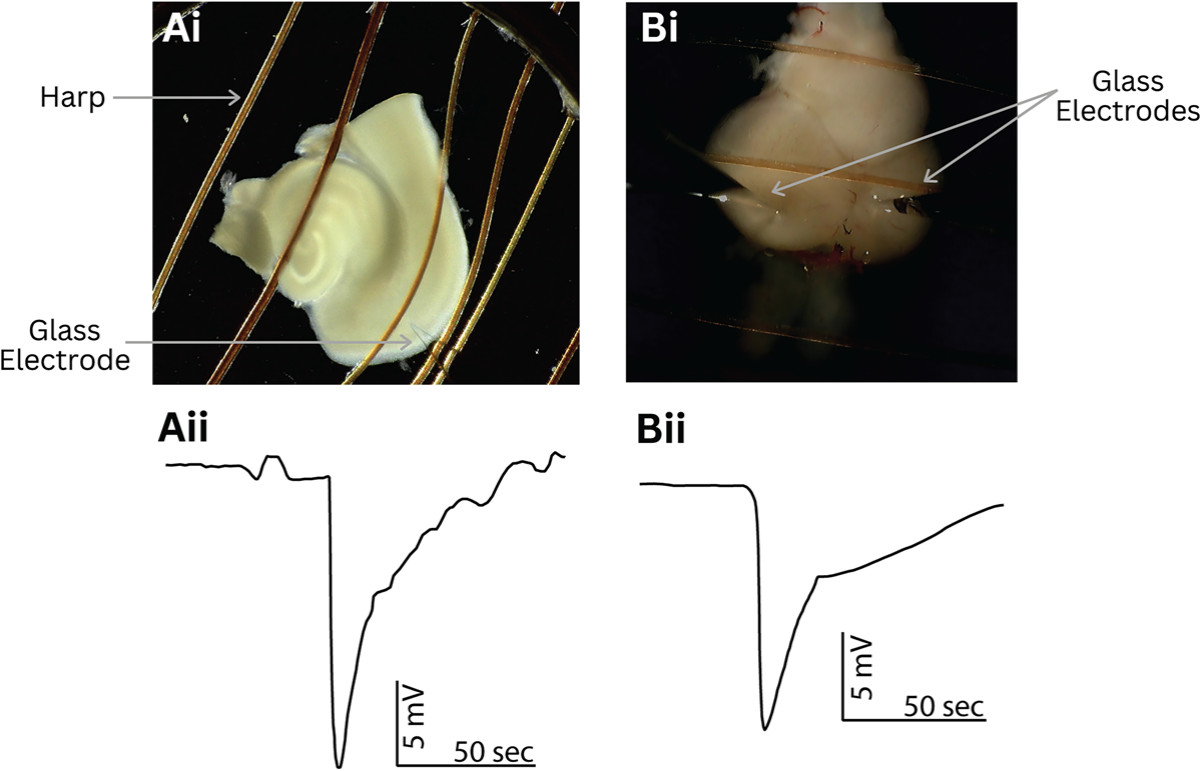
Representative data using 3D-printed holding chamber and stage with the mouse cortical tissue and zebrafish optic tectum. ***Ai***, Image showing a mouse neocortical and hippocampal tissue slice in the recording chamber secured by a platinum harp. The electrode placement in the neocortical tissue is shown. ***Aii***, Electrophysiological trace displaying a spreading depolarization in the mouse neocortical tissue induced with a high-potassium bath (12 mM) aided by a high-potassium microinjection (100 mM, ∼20 pl). ***Bi***, Image showing a zebrafish whole brain in a recording chamber secured by a harp. Electrode and picospritzer placement in the optic tectum is shown. ***Bii***, Electrophysiological trace displaying a spreading depolarization in the zebrafish optic tectum induced by high-potassium microinjection (100 mM, ∼20 pl).

All animal procedures were performed in accordance with the regulations of the Institutional Animal Care Committee and Brigham Young University Animal Care Committee. To perform experiments using this system (Fig. 5), the tissue samples were perfused with artificial cerebrospinal fluid (aCSF) at a rate of 3.4 ml/min using a peristaltic perfusion system (PPS2 Multi Channel Systems). One megaohm micropipettes were pulled from GC120TF-10 borosilicate glass (Harvard Apparatus), backfilled with aCSF solution [containing the following (in mM): 2 CaCl2; 1,341 MgCl2; 126 NaCl; 26 NaHCO3; 3.5 KCl; 1.26 NaH2PO4; 10 glucose], secured to a headstage, and placed in the optic tectum (zebrafish) or neocortex of the brain tissue samples, respectively, to obtain local field potential (LFP) recordings (Fig. 6*Ai*,*Bi*)*.* A chlorinated silver wire secured in the perfused recording solution served as a reference electrode. An inline heater was used to warm the solution to ∼36°C (SH-27B Harvard Apparatus and TC-344B, Warner Instrument Corporation). The direct current signal is unfiltered and amplified to a 10 times output with a custom-built amplifier then digitized with an NI USB-6341 X Series Multifunction DAQ board (National Instruments) and recorded using the WaveSurfer 1.0.6 software using MATLAB 2022b.

When conducting the outlined procedures, we found our 3D-printed recording stage and perfusion chamber to be an efficient way to successfully perform ex vivo electrophysiology recordings, maintaining tissue health and allowing for quick and easy modification to best suit our desired experiments. We collected representative data to indicate nervous tissue health over long periods and demonstrate experiment execution. We first induced a spreading depolarization in a rodent neocortical brain slice bathed in a high-potassium aCSF bath (12 mM) aided by a microinjection of high potassium (100 mM, ∼20 pl) after placement in our custom-made recording stage and chamber (Fig. 6*Ai*,*Aii*). Following that experiment, we induced spreading depolarizations in the zebrafish optic tectum by microinjection of high-potassium aCSF (∼20 pl at 100 mM) with a picospritzer (Parker Instrumentation) as demonstrated in Figure 6, *Bi* and *Bii*. Importantly, we were able to induce spreading depolarizations up to 2.4 h after placing the zebrafish brain in the recording chamber, demonstrating the ability of the system to support a live tissue for extended lengths of time*.* Both tissue preparations were also held in our custom holding chamber prior to experimentation (Fig. 5*A*). DC LFP recordings of spreading depolarizations in both ex vivo models were repeatably obtained using our 3D-printed system.

## Discussion

We introduce a versatile, 3D-printable design for a complete recording stage with interchangeable chambers. In stark contrast with commercially available options, such as the BSC1IS (AutoMate Scientific) and the BSC1 Brain Slice Heated Chamber (Digitimer), this system is easily accessible, affordable, adaptable, and completely customizable. Moreover, the recording stage and chambers have been demonstrated to maintain tissue health in ex vivo models long enough to conduct LFP experiments up to 2.4 h long.

Requiring nothing more than common laboratory materials, a 3D printer, and inexpensive PLA filament, this proposed design can easily be constructed anywhere in the world. Beyond reducing costs and enhancing customizability, it negates shipping time, known to be up to 6 months. With the included open-source STL files, the system components may be directly printed on a FDM printer without any additional modification. In this case, a Taz-6 3D printer (LulzBot) was used to print the parts. The customizable F3D designs are also provided (alterable using Autodesk Fusion360 or SolidWorks 3D CAD created by Dassault Systèmes), allowing researchers to modify any of the files to meet the specific needs of their lab. Once the components are printed, the assembly can be performed in <1 h by an individual completing the process for the first time.

As previously indicated, this proposed system is the only completely 3D-printable recording stage with interchangeable custom recording chambers (Fig. 2*D*). Published designs can support similar ex vivo experiments on a perfused tissue, but they are unable to do so independent of a commercial recording stage in contrast to our complete 3D-printed stage and recording chamber system (Slater et al., 2015). When examining the chambers themselves, customization grants this system capabilities not found in purchasable designs. Produced in minutes for well under a dollar, each recording chamber is designed to have the experimental solution directly perfused into the side of the recording chamber, as opposed to the bottom, eliminating the need for the typical flat, relatively level perfusion surface (Fig. 3*B*). However, the chamber can be modified to fit the researcher's preference for perfusion. This allows for complete control over the chamber fluid depth, surface material, and channel width and shape (and the corresponding circulation path), all of which are not possible to control on recording stages with a fixed design, regardless of what supporting tools are developed. Additionally, the chambers are identical in their outer rim shape (Fig. 1), ensuring an ideal fit when placed on the recording stage, a feature that affords researchers the ability to quickly exchange chambers and effectively conduct a variety of experiments throughout the day on the same system.

Significantly, the outlined ex vivo recording stage and submersion chamber have been found to successfully run diverse experiments. To demonstrate the effectiveness of this 3D-printed system, electrophysiological experiments were conducted on two disparate vertebrae ex vivo models: coronal rodent brain slices and whole adult zebrafish brains. As indicated by the extracellular LFP recordings (Fig. 6*Aii*,*Bii*), spreading depolarizations have been successfully stimulated in both animal models on our 3D-printed recording system, demonstrating the adaptability and efficacy of the newly proposed design. Accordingly, this model shows promise as a research platform for studying an assortment of biological tissues via ex vivo techniques, including research in long-term potentiation, synaptic connectivity (Pasinelli et al., 1995), and calcium imaging (Tada et al., 2014). In addition to collecting LFP data on rodent and zebrafish ex vivo models, the proposed 3D-printed system may be viable for experiments using cell cultures.

Moreover, with slight modifications, the system may be adjusted to accommodate research on other tissues that require different recording chamber specifications. For instance, narrowing of the recording chamber channel would increase perfusion flow, which can be advantageous with drug discovery research or preparations with higher carbogenation needs. For animal models larger than whole zebrafish brains, the chamber may be expanded and deepened. Adjustments to the recording chamber floor may also enable interface experiment capabilities.

Potential challenges with the constructed design include problems typical to structures created using additive fabrication methods (3D printing techniques), such as less resistance to impact when compared with thermoformed or injection-molded plastics (Komal et al., 2021). The result is that the design may wear quicker than commercially available plastic designs. Although fabricating the design using other materials (such as nylon, acrylonitrile butadiene styrene, or a mixed polymer) through additive manufacturing could improve the durability and longevity of the system, significantly higher energy requirements and potential health risks due to increased harmful emissions make doing so less practical (Milovanović et al., 2020; García and Pola, 2022; Warke and Puranik, 2022). Furthermore, because the system was only tested with LFP experiments, additional troubleshooting may be required when conducting other types of electrophysiological recordings to optimize the system for other functions. Moreover, the 3D-printed recording stage and recording chamber were designed to be used together, so the recording chamber will not fit on any other submersion or interface recording stage without considerable modification.

When considering the potential of FDM printers, it is no wonder that many research labs regularly produce custom parts using these additive fabrication techniques (Lücking et al., 2015; Salentijn et al., 2017). 3D printing is an effective method of reducing costs, increasing availability, offering increased customization, and expanding collaboration in research labs (Tropea et al., 2022; Quansah Amissah et al., 2023). With published open-source 3D printing designs for laboratory equipment and tools, researchers are able to utilize and build directly on the existing methods of others without having to recreate every step of the process (Michel et al., 2018). Such design collaboration may become an increasingly useful tool in the field of neuroscience as 3D-printable designs become increasingly available. Methods such as this will only expand our knowledge as we continue to produce novel ways to conduct high-quality, inexpensive research.

## Synthesis

Reviewing Editor: Timothy Jarome, Virginia Polytechnic Institute and State University Decisions are customarily a result of the Reviewing Editor and the peer reviewers coming together and discussing their recommendations until a consensus is reached. When revisions are invited, a fact-based synthesis statement explaining their decision and outlining what is needed to prepare a revision will be listed below. The following reviewer(s) agreed to reveal their identity: Robert Mans.

The manuscript solves a significant problem faced by electrophysiologists in resource-limited laboratories in a flexible and accessible manner. The new technology could be applied by researchers using several types of animal models/tissues. The flexibility, simplicity and cost-effectiveness of the technology provides the potential for large-scale use in a teaching laboratory in addition to research.

However, more details about the local field potential (LFP) recording procedure would be beneficial. More specifically, were the LFPs evoked or spontaneous? This detail should be incorporated into the Results and corresponding figure legend. If the LFPs were evoked, a very brief description of the stimulation protocol would add value to the manuscript.
